# Prevalence and molecular characterisation of *Eimeria* species in Ethiopian village chickens

**DOI:** 10.1186/1746-6148-9-208

**Published:** 2013-10-15

**Authors:** Lisa Luu, Judy Bettridge, Robert M Christley, Kasech Melese, Damer Blake, Tadelle Dessie, Paul Wigley, Takele T Desta, Olivier Hanotte, Pete Kaiser, Zelalem G Terfa, Marisol Collins, Stacey E Lynch

**Affiliations:** 1 Institute of Infection and Global Health, University of Liverpool, Leahurst Campus, Liverpool CH64 7TE, United Kingdom; 2 International Livestock Research Institute, Addis, Ababa, Ethiopia; 3 Debre Zeit Agricultural Research Centre, Ethiopian Institute of Agricultural Research, Debre Zeit, Ethiopia; 4 Department of Pathology and Pathogen Biology, Royal Veterinary College, North Mymms AL9 7TA, UK; 5 Ecology and Evolution, School of Life Sciences, University of Nottingham, University Park, Nottingham NG7 2RD, UK; 6 The Roslin Institute and Royal (Dick) School of Veterinary Science, University of Edinburgh, Midlothian EH25 9RG, UK; 7 Management School, University of Liverpool, Liverpool L69 7ZH, United Kingdom

**Keywords:** Eimeria, Coccidiosis, Village chickens, Ethiopia, Molecular characterisation

## Abstract

**Background:**

Coccidiosis, caused by species of the apicomplexan parasite *Eimeria*, is a major disease of chickens. *Eimeria* species are present world-wide, and are ubiquitous under intensive farming methods. However, prevalence of *Eimeria* species is not uniform across production systems. In developing countries such as Ethiopia, a high proportion of chicken production occurs on rural smallholdings (i.e. 'village chicken production’) where infectious diseases constrain productivity and surveillance is low. Coccidiosis is reported to be prevalent in these areas. However, a reliance on oocyst morphology to determine the infecting species may impede accurate diagnosis. Here, we used cross-sectional and longitudinal studies to investigate the prevalence of *Eimeria* oocyst shedding at two rural sites in the Ethiopian highlands.

**Results:**

Faecal samples were collected from 767 randomly selected chickens in May or October 2011. In addition, 110 chickens were sampled in both May and October. *Eimeria* oocysts were detected microscopically in 427 (56%, 95% confidence interval (95% CI) 52-59%) of the 767 faecal samples tested. Moderate clustering of positive birds was detected within households, perhaps suggesting common risk factors or exposure pathways. Seven species of *Eimeria* were detected by real time PCR in a subset of samples further analysed, with the prevalence of some species varying by region. Co-infections were common; 64% (23/36, 95% CI 46-79%) of positive samples contained more than one *Eimeria* spp. Despite frequent infection and co-infection overt clinical disease was not reported. *Eimeria* oocysts were detected significantly more frequently in October (248/384, 65%, 95% CI 60-69%), following the main rainy season, compared to May (179/383, 47%, 95% CI 42-52%, p < 0.001). *Eimeria* oocyst positivity in May did not significantly affect the likelihood of detecting *Eimeria* oocyst five months later perhaps suggesting infection with different species or immunologically distinct strains.

**Conclusions:**

*Eimeria* spp oocysts may be frequently detected in faecal samples from village chickens in Ethiopia. Co-infection with multiple *Eimeria* spp was common and almost half of Eimeria positive birds had at least one highly pathogenic species detected. Despite this, all sampled birds were free of overt disease. Although there was no evidence of a difference in the prevalence of oocysts in faecal samples between study regions, there was evidence of variation in the prevalence of some species, perhaps suggesting regional differences in exposure to risk factors associated with the birds, their management and/or location-specific environmental and ecological factors.

## Background

Indigenous chickens kept under village, scavenging production systems represent 97% of the chickens farmed in Ethiopia [1]. In this system, micro- and macro-parasitic infectious diseases, combined with limited extension and veterinary services, are major constraints to village chicken production. Poor management and limited resources hinder the development of chicken production by reducing outputs and by predisposing to disease outbreaks [2-4].

Coccidiosis, caused by species of the apicomplexan parasite *Eimeria,* is a major cause of mortality and morbidity in chickens worldwide, but clinical manifestations are not uniform across production systems. Endemic infections and long term exposure without treatment in broiler production systems can result in reduced feed conversion efficiency and growth rate, and in extreme cases lead to tissue damage in the intestinal tract, promoting secondary infections with opportunistic pathogens [5,6].

Coccidiosis is reported to be a major disease impacting Ethiopian village chickens [7,8]. *Eimeria* oocysts have been detected in up to 61% [8] of faecal samples collected from indigenous village chickens in central Ethiopia, with six of the seven valid *Eimeria* species known to infect the chicken identified by microscopy [7-9]. However, the advent of molecular techniques has demonstrated that microscopy and post mortem identification of *Eimeria* parasites can lead to incorrect identification of parasites [10,11]. The correct speciation of parasites is important due to the immunological and pathogenic variation across the genus [10-12]. Microscopy was therefore carried out to determine the prevalence of *Eimeria* genus parasites in indigenous village chickens in two regions of Ethiopia, complimented by molecular characterisation to identify the *Eimeria* species circulating.

## Results and discussion

### Detection of *Eimeria* oocysts in faecal material

Of the 767 faecal samples examined microscopically, *Eimeria* oocysts were detected in 427 (56%: 95% confidence interval (95% CI) 52-59%). The prevalence reported in this study is comparable to the 42.2% (95% CI 31-55%: our calculation) previously reported in Ethiopian highland areas [7]. The detection of *Eimeria* oocysts was significantly higher in October (248/384, 65%, 95% CI 60-69%) compared to May (179/383, 47%, 95% CI 42-52%; chi-square p-value <0.001). No significant differences were found in prevalence at region or village levels. All faecal samples detected positive for *Eimeria* oocysts were collected from chickens with no overt signs of clinical diseases. As birds were randomly selected from within the household flock and presence of clinical disease was not a criterion for bird selection, the absence of overt disease in our sample suggests that the prevalence of clinical coccidiosis is rare compared to the prevalence of *Eimeria* carriage. Furthermore, the high prevalence of oocyst shedding detected in apparently healthy birds is strong evidence that the detection of oocysts alone in chicken faecal samples should not lead to a definitive diagnosis of coccidiosis. Quantitative identification to the species level is clearly required to account for species-specific pathogenicity.

There was evidence of moderate clustering of oocyst positivity in faecal samples collected from chickens within a single family-flock. Estimates of intraclass clustering coefficient (ICC) for household indicated 18.3% (using binary linearization method) or 13.4% (using the latent-variable method) of the variation in the presence/absence of oocysts is due to variation within households. These values are consistent with those reported for some “highly contagious” infections by Otte & Gumm [13], such as Newcastle disease (ICC 18%), but are lower than those for other “highly contagious” infections, including infectious bursal disease (37%) and bovine viral diarrhoea (23% to 42%). Interestingly, they are somewhat similar to the ICC values reported for *Eimeria* spp in cattle (18% to 30%). Hence, the ICC estimates in this study suggest that although the bulk of the variation in detection of oocysts occurs because of variation within birds, evidence of clustering of positive birds within households may suggest a role for exposure to particular risk factors for birds within household flocks – identification of these may suggest useful targets for control interventions. Further analysis found no evidence of clustering at higher spatial scales (i.e. village or region). However, molecular characterisation revealed species-level variation between regions (see below). Hence, while there was no evidence of a difference in the prevalence of oocysts in faecal samples between regions, there was evidence of variation in the prevalence of some species, perhaps suggesting regional differences in exposure to in risk factors associated with the birds, their management and/or location-specific environmental and ecological factors.

In addition to the repeat cross sectional study, faecal samples were collected from 110 chickens during both sampling periods. *Eimeria* oocysts were detected in the faeces of 32 (29%: 95% CI 21-39%) chickens at both time points, 63 (57%, 95% CI 47-67%) at one time point (23 were found positive only in May, whereas 40 were positive only in October) and 15 (14%; 95% CI 8-22%) at neither of the time points. This finding is consistent with prolonged sub-clinical re-infection (with the same or different species, or immunogically distinct strains) in almost one-third of the birds. Within this subset of samples, the prevalence of *Eimeria* oocysts was greater following the wet season in October (72/110, 65%, 95% CI 56-74%) compared to May (55/110, 50%, 95% CI 41-59%; McNemar chi-square p-value = 0.04) and this is in line with similar observations described for *Eimeria* in commercial chickens in Pakistan [14]. The prevalence of *Eimeria* oocysts in this subset in October was not significantly different to that of the randomly selected birds at the same time (chi square p-value = 0.7). The prevalence of *Eimeria* oocysts in the October sample for birds that were negative in May was 73% (40/55; 95% CI 59-83%) compared to 58% (32/55; 95% CI 44-71%) for birds detected positive in May. However, this difference was not statistically significant (Chi square p-value = 0.2). This may imply that previous detection of oocysts does not impact the likelihood of detecting oocysts five months later, perhaps suggesting infection with different species or immunologically distinct strains.

### Molecular characterisation of circulating *Eimeria* species

Eimerian genomic DNA was detected in 47 of the 56 samples tested (Horro 22/26 and Jarso 25/30). Seven species of *Eimeria* were detected (Table 1). Regional comparisons show differences in the predominant circulating species; *Eimeria praecox* was found in 95% (21/22, 95% CI 75-99%) of samples tested from Horro and *E. maxima* was found in 60% (15/25, 95% CI 39-78%) of samples tested from Jarso. *Eimeria praecox* is widely considered to be among the least pathogenic *Eimeria* species that infect the chicken, although recent reports of a pathogenic isolate illustrates the risk posed by such a high prevalence of this species [15]. The high prevalence of *E. maxima* in Jarso may be relevant to the birds found to be positive on both sampling occasions since, while highly immunogenic, this species is commonly characterised by strain-specific immunogenicity [16]. There is evidence in the data to support this hypothesis; previous infection (as detected by faecal microscopy) appeared to have no protective effect in Jarso, with prevalence in October of 65% (45-80%) among birds negative in May compared to 58% (39-74%) for those positive in May (chi square p-value = 0.8). Whilst still not statistically significant, there was some evidence of a greater effect of past infection in Horro, where *E. praecox* predominated, with a prevalence in October of 83% (62-95%) among birds negative in May compare to 59% (37-79%) for those positive in May (chi square p = 0.1). *Eimeria mitis* was only found in samples collected from Jarso (28%, 7/25, 95% CI 14-48%). Although the haemorrhagic species considered to be highly pathogenic were not the most frequently isolated species (i.e. *E. tenella*, *E. necatrix* and *E. brunetti*[17]), highly pathogenic species were detected in almost half of all samples (20/47, 43%, 95% CI 29-58%).

**Table 1:** **Frequency of detection of*Eimeria*species in chicken faecal samples**

	**Region**			**Mixed species infection (n = 23)**	**Single species infection (n = 13)**
**Species**	**Horro**	**Jarso**	**Fisher’s exact p-value**		
	**(n = 22)**	**(n = 25)**			
*E. acervulina*	9 (40%)	10 (40%)	0.8	12 (52%)	1 (8%)
*E. brunetti*^1^	4 (18%)	5 (20%)	0.9	5 (22%)	0 (0%)
*E. maxima*	5 (22%)	15 (60%)	0.02^2^	13 (57%)	4 (31%)
*E. mitis*	0 (0%)	7 (28%)	0.01^2^	2 (9%)	2 (15%)
*E. necatrix*^1^	4 (18%)	2 (8%)	0.4	4 (17%)	0 (0%)
*E. praecox*	21 (95%)	9 (36%)	<0.001^2^	17 (74%)	6 (46%)
*E. tenella*^1^	3 (12%)	7 (28%)	0.3	3 (13%)	0 (0%)

Samples were collected from 47 indigenous chicken faecal samples in two regions of Ethiopia (Horro and Jarso). The frequency of detection of multiple and single *Eimeria* species infections among 36 of these 47 which were each known to come from a single bird are also presented.

^1^Considered to be highly pathogenic [17].

^2^Significantly different (p <0.05).

**Figure 1:**
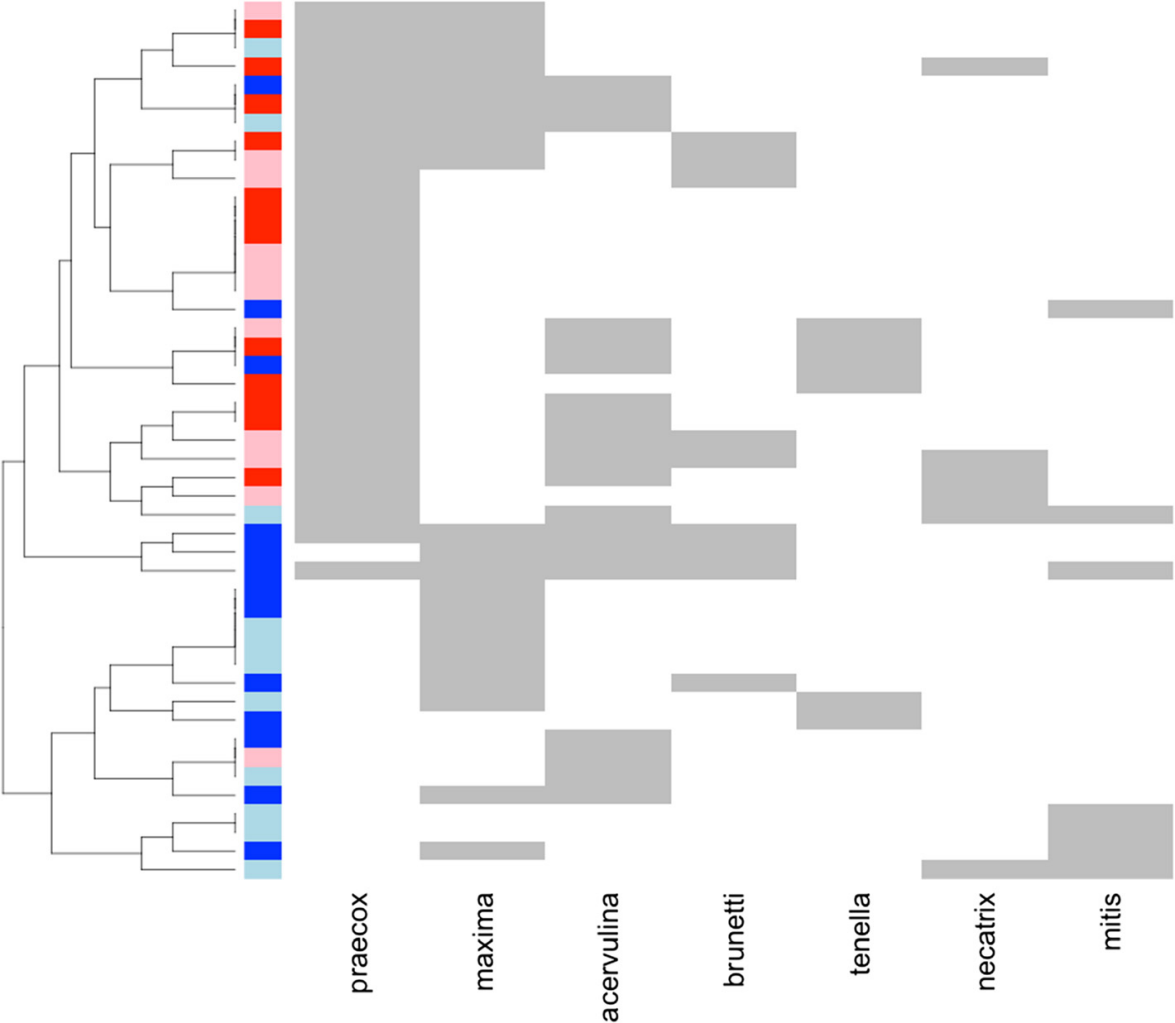
**Co-occurrence of *Eimeria *spp.** Faecal samples were collected from 47 chickens from two regions of Ethiopia in May 2011. Each row represents one sample. Red boxes indicated samples from Horro, blue indicate samples from Jarso. Light and dark shading indicates each of the pairs of villages within each region.

Of the 47 samples where *Eimeria* oocysts were detected, 36 were known to be collected from individual birds, the rest included fresh chicken faeces sampled from the environment and may have included faeces from more than one bird. Of these 36 samples, more than one *Eimeria* spp was detected in 64% (23/36, 95% CI 46-79%). Species frequencies in co-infection are presented in Table 1. *Eimeria praecox* was the most common species and detected in 46% of single infections and 74% of co-infections in this study. The frequency of species presence in mixed infections was strongly correlated with overall prevalence of that species (Spearman r = 0.82, p = 0.03) suggesting that there was no species predilection for co-infection, except that due to prevalence. There was evidence for spatial clustering of patterns of co-infection within each of the two study regions (Figure 1), however, this was less obvious when *E. mitis* and *E. praecox* were excluded from the analysis (data not shown), suggesting much of the spatial variation in patterns of co-occurrence was due to the different prevalence of *E. mitis* and *E. praecox* between the two regions.

In this study, the isolation of *Eimeria* oocysts was not associated with overt clinical disease. However, *Eimeria* species with a high pathogenic potential (e.g. *E. tenella, E. necatrix, E. brunetti*[17] were detected. The circulation of pathogenic *Eimeria* species may significantly contribute to high mortality in chickens less than eight weeks of age [17]. The mortality rate among young birds is high in Ethiopian village production systems (estimated at around 60% [3]) but the role of *Eimeria* species in this mortality is yet to be determined. Furthermore, where additional contributory factors co-occur, such as malnutrition or co-infection with other pathogens, even low oocyst infections may cause significant disease (e.g. co-infection with *Clostridium perfringens*, causing Necrotic Enteritis). Although several *Eimeria* control measures, such as vaccines and anti-coccidial drugs, are available in developing countries, these may not be suitable for low-input rural poultry production systems. Changes in the management of poultry and improved nutrition are, however, recognised as economical and effective ways to reduce the adverse effects of non-viral diseases. The birds in this study were kept in a variety of ways, but the majority were housed either within the family dwelling or the kitchen building at night, where they would perch on the rafters or partitions, or on the floor. Purpose-built housing or perches were rarely seen, although most households provided a nest box for hens with chicks. Removal of faeces was done as part of daily household cleaning, and these were used by some households for crop fertiliser. Practical ways to reduce disease transmission could include improving hygiene measures such as prevention of overcrowding, disposal of sick and dead birds and regular removal of excreta, provision of improved housing with elevated perches for adult birds and separate overnight shelter and feed provision for chicks from older birds [18,19].

## Conclusion

In conclusion, *Eimeria* spp oocysts may be frequently detected in faecal samples from village chickens in Ethiopia in the absence of overt clinical signs. Furthermore, while there was no evidence of a difference in the prevalence of oocysts in faecal samples between regions, there was evidence of variation in the prevalence of some species, perhaps suggesting differences in risk factors associated with the birds, their management and/or location-specific environmental and ecological factors. Importantly, we identified *Eimeria* species that have high pathogenic potential circulating in apparently healthy adult Ethiopian indigenous village chickens. Further investigation of the role of *Eimeria* spp in both clinical disease and subclinical effects on production are required to better understand their impact and whether interventions to reduce the burden of these organisms in village poultry may offer benefits, or even impact negatively by disrupting endemic stability.

## Methods

### Ethics statement

This study was approved by the University of Liverpool Committee on Research Ethics (reference RETH000410). Participants were provided with verbal information to inform them of the purpose of the study, that participation was entirely voluntary, that they were free to leave the study at any time and that all data would be kept securely. Verbal informed consent was obtained prior to collection of data and samples. Verbal information and verbal informed consent was deemed appropriate due to the expected high illiteracy rate among participants, many of whom would therefore be unable to understand written information or consent forms and who may be unable provide a written consent. The consent procedure was approved by the University of Liverpool Committee on Research Ethics and documented for each participant by a tick box on the information sheet that was read to each potential participant and which was ticked in the presence of the participant.

### Sample collection

Two highland regions were selected for this study; Horro district (Western Ethiopia, villages surrounding Shambu located at 9°34′N 37°06′E with elevations ranging from 2255 to 2613 meters above sea level) and Jarso district (Eastern Ethiopia, villages surrounding Ejersa Goro located at 9°29′N 42°14′E with elevations ranging 1993 to 2719 meters above sea level). Poultry production in the study areas predominantly used local ecotypes and is based on a free-ranging scavenging system. The median flock size in Horro was 8 (interquartile range 4.0 to 14.5) and in Jarso was 4 (2.75 to 6.0). The birds were kept for sale (of birds and eggs), occasional home consumption and for socio-cultural purposes. Within each region, two pairs of neighbouring villages were selected for sampling, based on their willingness to participate, and on knowing there was no recent flock improvement programme in the area; and in each village 25 households were randomly selected from lists of households maintained by local agricultural offices. Faecal samples were collected from two randomly-selected birds within each household. Only birds estimated to be greater than 6 months of age and of the local ecotype, based on owner reporting and physical examination, were eligible for inclusion in the study.

Faecal samples were collected from study sites in May 2011 (n = 383) and October 2011 (n = 384), months flanking the main Ethiopian rainy season between July and September. Where possible, birds sampled in May were also resampled in October, resulting 110 additional samples in October. The identities of the resampled chickens were confirmed by comparing a series of photographs (resolution of 96 dpi or greater), including lateral, dorsal and frontal views of the bird and close-up views of the head and feet, taken on both occasions.

### Microscopic detection of *Eimeria* oocysts

Concentration McMaster salt flotation technique was used to determine the prevalence of *Eimeria* genus parasites within the study chicken population [20].

### Molecular characterisation of *Eimeria* oocysts

To determine the species of *Eimeria* circulating at these two sites, positive samples were randomly selected for further molecular analysis. Oocysts were isolated [21] from between 0.60 grams and 17.09 grams of faecal matter; DNA was extracted using a Qiagen QIAmp stool extraction kit according to the manufactures instructions. Extracted DNA samples were tested by real time PCR, as described by Vrba et al. [22].

### Statistical analysis

The proportion of samples found positive (by microscopy) in each region and village, and at each sampling round were initially compared using chi square tests. Subsequently, multilevel binary logistic regression models were constructed with the outcome defined as positive or negative based on microscopy and individual birds as level 1 and households as level 2. Intercept only models were used to estimate the proportion of variation due to the household level by calculating the intraclass clustering coefficient (ICC) using two methods: binary linearization and the latent-variable method [23]. For the birds which underwent repeat sampling, McNemar’s test was used to compare the proportions positive at each time point, chi square tests were used to compare the proportions positive in October among birds that were either positive or negative at the May sampling and Fisher’s exact test was used to compare the frequency of each species between the two study regions.

For each *Eimeria* species, the frequency of presence in mixed infections was compared to its overall prevalence using Spearman rank-order correlation. Co-occurrence of *Eimeria* species was evaluated using cluster analysis using the Manhattan distance measure and McQuitty agglomeration method. All statistical analyses were performed using R (http://www.R-project.org/).

## Abbreviations

CI: Confidence interval; ICC: Intraclass clustering coefficient; PCR: Polymerase chain reaction.

## Competing interests

The authors declare that they have no competing interests.

## Authors’ contributions

LL, RMC and SEL designed the study, analysed and interpreted the data and, with JB, wrote the manuscript. LL, JB, TD, DB, ZT, KM, MC and SEL assisted with fieldwork and/or laboratory analysis. LL, JB, RMC, DB, TD, PW, TTD, OH, PK, ZT, MC and SEL helped draft and review the manuscript and contributed intellectually to the development of the research. All authors read and approved the final manuscript.
